# Clustering on Human Microbiome Sequencing Data: A Distance-Based Unsupervised Learning Model

**DOI:** 10.3390/microorganisms8101612

**Published:** 2020-10-20

**Authors:** Dongyang Yang, Wei Xu

**Affiliations:** 1 Division of Biostatistics, Dalla Lana School of Public Health, University of Toronto, Toronto, ON M5T 3M7, Canada; dongyang.yang@mail.utoronto.ca; 2 Department of Biostatistics, Princess Margaret Cancer Centre, Toronto, ON M5G 2M9, Canada

**Keywords:** clustering, microbiome, unsupervised learning, high-dimension

## Abstract

Modeling and analyzing human microbiome allows the assessment of the microbial community and its impacts on human health. Microbiome composition can be quantified using 16S rRNA technology into sequencing data, which are usually skewed and heavy-tailed with excess zeros. Clustering methods are useful in personalized medicine by identifying subgroups for patients stratification. However, there is currently a lack of standardized clustering method for the complex microbiome sequencing data. We propose a clustering algorithm with a specific beta diversity measure that can address the presence-absence bias encountered for sparse count data and effectively measure the sample distances for sample stratification. Our distance measure used for clustering is derived from a parametric based mixture model producing sample-specific distributions conditional on the observed operational taxonomic unit (OTU) counts and estimated mixture weights. The method can provide accurate estimates of the true zero proportions and thus construct a precise beta diversity measure. Extensive simulation studies have been conducted and suggest that the proposed method achieves substantial clustering improvement compared with some widely used distance measures when a large proportion of zeros is presented. The proposed algorithm was implemented to a human gut microbiome study on Parkinson’s diseases to identify distinct microbiome states with biological interpretations.

## 1. Introduction

Human microbiome carries complicated relationships among species yet profoundly connect with human health. Studies to understand of the effects of the microbiome on human diseases have been conducted recently. For example, evidence linking Parkinson’s disease to the gut microbiome is presented in Hill-Burns’ work [1]. The microorganisms in the human body consist of over 1000 species of bacteria [2,3]. Modern technology promotes the scope of microbiome research so that the massive microbiome data can be generated by 16S rRNA sequencing or shotgun metagenomic sequencing.

The microbiome data that is generated by the sequencing technology needs to be classified into taxonomic groups. The abundance of a species is quantified based on their similarity to operational taxonomic units (OTUs) and formed into discrete counts of sequence reads. Typical reads have excessive zeros due to either sampling errors or their unique features that only dominant microorganisms are shared among samples. Positive reads are usually skewed with extreme sparse count measures, which are called overdispersion. Traditional statistical methodologies encounter challenges in microbiome studies and result in potential bias [4,5,6].

An essential research question of microbiome study is to determine whether the microbiota can be stratified into subgroups. If so, how many groups are there, and how to interpret the strata, i.e., does the classification differentiate treatments, diseases, or genetic types. To answer these questions, the measurement of similarity between two microbial communities is desirable. Beta diversity has been proposed to fit diverse purposes, providing various results in assessing the differences between communities. For microbial composition, beta diversity measures the distance among communities based on measurement abundance, either observed counts or relative abundance, calculated based on a dissimilarity or distance measure to quantify the similarity between samples. Many non-parametric statistical methods have been developed to quantify distance measures. For instance, Euclidean and Manhattan distances are most commonly used. Other beta diversity metrics, such as Bray-Curtis Distance (BC) [7], Jensen-Shannon Distance (JS), Jaccard Index, UniFrac distances (unweighted [8], weighted [9], and generalized [10]) are also frequently applied in microbiome studies. Besides the distance metrics, graphical network models have also been introduced in Sparse Inverse Covariance Estimation for Ecological Association Inference (SPIEC-EASI [11,12]). The method applied a centered log-ratio transformation to the OTU data followed by either neighborhood selection or sparse inverse covariance selection to estimate the interaction graph. However, the method encounters difficulty in the underdetermined data regime. For example, the number of OTUs is much larger than the number of samples. In addition, SPIEC-EASI method relies on a single variance-covariance matrix which may not be able to completely recover the underlying OTU network due to the complex structure of the microbial community. In comparison, the mixture models are more flexible from the way of construction, and it may approximate the real distribution of taxa and lead to more accurate estimations of distances that are used in clustering.

The clustering of microbiome samples has been achieved in many studies using a variety of approaches. Clustering algorithms, including distance-based and parametric modeling, have been used to group subjects according to the microbiome samples. Two main types of distance-based approaches are hierarchical clustering [13,14,15] with different linkage options and discrete clusterings such as k-means [16,17,18,19] and Partition Around Medoids (PAM) [20,21,22,23]. Discrete clustering requires a pre-specified number of clusters while different linkages for hierarchical clustering such as Ward linkage, complete linkage, simple linkage, and average linkage provide rules to agglomerate. The Dirichlet-multinomial regression model [24] is the most frequently used on microbial metagenomic data for model-based clustering. Extensions such as the Sparse Dirichlet-multinomial regression technique [25] and finite mixtures of the Dirichlet-multinomial model [26,27] have been proposed to improve on different statistical aspects. These regression models investigate the relation between microbiome composition data and environmental or biological factors. However, currently, only a univariate analysis could be performed. On the other hand, distance-based clustering allows us to take multiple OTUs into account simultaneously.

For the clustering algorithms, it is critical to determine the optimal number of clusters *K*. Therefore, validation measures for clustering have been explored to identify the ideal number of groups *K* to represent data [28,29,30,31,32]. Validation indices are used to measure the quality of a clustering result in two ways: internal and external. An internal validation index is to use the information from the data only to decide the optimal number of clusters, such as the Silhouette width index [33], prediction strength [34], Calinski-Harabasz index [35], and Laplace approximation. Validation scores can be computed for different *K*, respectively, and then they identify the optimal *K* accordingly. An external validation index, on the other hand, uses prior knowledge to compare the predictive results.

There are different combinations of distance measures and algorithms. Koren et al. [23] computed the distances with and without the root square of the JS, BC, weighted and unweighted UniFrac distances and selected the number of clusters with the prediction strength and the silhouette index used in the PAM algorithm. Unlike Koren’s approach, Hong et al. [14] applied K-means with Euclidean distance to identify two clusters, in which they believe the number of clusters makes biologically sense in their study. It is noticeable that no standard clustering pipelines are available, and therefore the various approaches to the recognition of subgroups lead to widely different results. This phenomenon is more evident for microbiome datasets due to the features of microbial data—overdispersion and excessive zeros, which will cause more variations in the process of gathering microbiome into groups.

We develop an innovative clustering approach taking a mixture distribution, rather than a beta diversity metric, as the distance measure and applying a clustering algorithm to the microbiome data to characterize sub-populations. The algorithm also involves selecting the optimal number of clusters based on chosen internal indices, and the results are compared between several distance measures and different evaluation methods. The performance of the proposed algorithm is evaluated through comprehensive simulation studies and a real human gut microbiome dataset on Parkinson’s diseases.

## 2. Materials and Methods

A mixture model is a probabilistic model for representing subpopulations within an overall population, which are frequently used in unsupervised learning [36,37,38,39]. Simple distributions such as Binomial, Poisson, and Gaussian are occasionally unable to model more complex data. For instance, microbiome data may consist not only one mode (zeros and low counts), high probability mass for larger counts, and smaller probability mass for high counts. In this case, the data is better to be modelled in terms of a mixture of several components, where each component is a simple probabilistic distribution.

To deal with the unique characteristics of microbiome data—sparsity with abundant zeros, we incorporate a mixture model proposed by Shestopaloff [40] to attain the beta diversity measures for partition. The mixture model focuses on the distribution of a single OTU across a population which can address the problem of sparsity between samples. It parametrically models the counts’ underlying rate distribution, including low counts OTUs and extremely high counts. For pairwise distances between individual samples, the formulated mixture’s probability is used in $L_{2}$ norms distances.

By using such a model, the beta diversity measure contains information regarding zero part in the data and distinguishes between the structural and sampling zeros. The proposed mixture models assume the observed counts are from a Poisson distribution with individual-specific rates, and the rates are sampled from some general population distribution, which can be approximated by a set of mixture components. Conditional on the estimated population rate distribution, the subject-specific rate distribution is estimated through individual mixture distribution given the observed sample counts and resolution. After that, beta diversity measure can be calculated by assessing the pairwise differences between samples for a particular OTU using the individual mixture distribution.

### 2.1. Mixture Model

To introduce some notations for the following section, let $n_{ij}$ be the number of times an OTU was observed from a sample, where *i* is the subject, i=1,…,I and *j* is the OTU, j=1,…,J. Resolution $N_{i}$ is the sum of the total reads of an individual; thus, it is defined as $N_{i}=\sum_{j}n_{ij}$. To connect the general population distribution to the collected data, the rates can be scaled by the average total reads ${\bar{N}}_{j}$ for OTU *j*, ${\bar{N}}_{j}=\sum_{i}N_{ij}/I$. Therefore, the relative resolution $t_{ij}$ is defined as $t_{ij}=N_{ij}/{\bar{N}}_{j}$.

The mixture model consists of five components to accommodate the complexity of microbiome data. For individuals who are never disclosed to OTU *j*, the model assigns a zero point mass $P(n_{ij}=0)=1$. For the rates close to zero, a set of adjacent left-skewed distributions with consecutive parameters is used to represent the low rates. For larger rates, the model accommodates a set of Gamma distribution with parameters which are all integers and are derived from the posterior of the Poisson rate λ given an observed count *n*, that is λ|n∼Γ(n+1,1). For higher counts that are less dense, the parameters are defined by truncating the interval uniformly after transforming the data range by a log scale. Lastly, for the even higher counts which are too sparse, the model selects a sufficiently large cut-off point and combines all the observations greater than that point into a high point mass $P(n_{ij}>C)=1$.

The parts other than zeros and extreme high point masses consist of several components with fixed parameters from Gamma distributions. Since each OTU’s distribution is estimated independently, we target one OTU per time and drop the subscript *j* onward. Define the estimated weights for each mixture components described above as $\vec{w}=\left(w_{z},w_{1},\ldots ,w_{M},w_{h}\right)$ where $w_{z}$ is the weight for zero point mass, $w_{m}$ where m=1,…,M is the weight for the Gamma components, and $w_{h}$ is the weight for the high point mass. Weights estimation is utilized to compose the final mixture model and can be calculated by minimizing the squared differences between the observed aggregated counts and the expected ones. For a particular OTU, the observed aggregated counts can be expressed as $y_{k}=\sum_{i}I\left(n_{i}=k\right)$, where *k* is the number of counts observed in a sample. The expected aggregated counts are the probability of observing *k* counts from each mixture component in a sample. For the Gamma components, counts are distributed in negative binomial distribution $NB(\alpha_{m},\frac{\beta_{m}}{t_{i}+\beta_{m}})$ conditioned on the relative resolution $t_{i}$. Define the probability of observing a count *k* from the *m*th mixture component conditional on $t_{i}$ is $p_{kmi}=P_{NB}\left(K=k|\alpha_{m},\beta_{m},t_{i}\right)$. The expected aggregate counts ${\hat{y}}_{k}$ from all mixture components is

(1) $$\begin{array}{cc} {\hat{y}}_{k} & =\sum_{m}{\hat{y}}_{km}\\ \; & =\sum_{m}w_{m}\cdot p_{km}\cdot I \end{array}$$

The estimate of weights $\vec{w}$ is obtained by optimizing the objective function

(2) $$argmin_{\vec{w}}\sum_{k\in \vec{k}}{\left[y_{k}-\sum_{w_{m}\in \vec{w}}w_{m}\cdot p_{km}\cdot I\right]}^{2}$$

$$s.t.\sum_{m}w_{m}=1,\;w_{m}\geq 0,\forall m$$

and using bootstrap replicates to find an optimal set of models as mixture components. Details of the bootstrap approach can be found in Appendix A.

For each subject *i*, the probability density function (PDF) of the mixture model is defined as the product of the individual-specific mixture weights and the count probability from mixture components

(3) $$\begin{array}{cc} P_{i} & =[P_{i}\left(z\right),P_{i}\left(0\right),\ldots ,P_{i}\left(C\right),P_{i}\left(C+\right)]\\ \; & ={\vec{w}}_{i}^{T}\cdot \left[P\left(z\right),P\left(0\right),\ldots ,P\left(C\right),P\left(C+\right)\right], \end{array}$$

where

$$P\left(k\right)=[P_{G_{z}}\left(k\right),P_{G_{1}}\left(k\right),...,P_{G_{M}}\left(k\right),P_{G_{h}}\left(k\right)],$$

and

$$P_{i}\left(z\right)=w_{G_{z}},$$

$$P_{i}\left(h\right)=1-\sum_{k=1}^{C}P_{i}\left(k\right)-w_{G_{z}}$$

### 2.2. Distance Measures

#### 2.2.1. $L_{2}$ Norms Distances

After finalizing the mixture model distribution, distance measures can be calculated through the pairwise distances between samples using probability distribution. Three distance measures based on $L_{2}$ norms are considered for comparisons: discrete $L_{2}$ PDF norms ($L_{2}$-D PDF), discrete $L_{2}$ CDF norms ($L_{2}$-D CDF), and continuous $L_{2}$ CDF norms ($L_{2}$-C CDF).

Given a mixture distribution’s PDF as $P_{i}$ and its cumulative density function (CDF) as $F_{i}$ for subject *i*, the distance of discrete $L_{2}$ PDF norms are computed by

(4) $$\begin{array}{cc} D_{L_{2}-D,PDF}\left(i,j\right) & =∥P_{i}-P_{j}{∥}^{2}\\ \; & ={\left[P\left(k\right)\right]}^{2}\sum_{q\in \vec{q}}{\left(w_{i}-w_{j}\right)}^{2} \end{array}$$

for i,j=1,…,I and i≠j, where $\vec{q}={\left(z,0,1,...,M,h\right)}^{\prime }$. Similarly, the distance of discrete $L_{2}$ CDF norms can be calculated analogously using the cumulative density function instead of the PDF.

The continuous $L_{2}$ CDF norms can be calculated based on the CDF of two individual-specific mixture models. The $L_{2}$-C CDF norms can be computed by

(5) $$\begin{array}{cc} D_{L_{2}-C,CDF}\left(F_{i},F_{j}\right) & =\int_{0}^{C}{\left[F_{i}\left(k\right)-F_{j}\left(k\right)\right]}^{2}dk\\ \; & ={\left(w_{i}-w_{j}\right)}^{T}G_{q_{1},q_{2}}\left(w_{i}-w_{j}\right) \end{array}$$

where $G_{q_{1},q_{2}}$ is a matrix such that $\int_{0}^{C}G_{q_{1}}\left(k\right)G_{q_{2}}\left(k\right)dk$ represents the two components $\left(q_{1},q_{2}\right)$ in the mixture model. See details of the derivation in [40].

#### 2.2.2. Other Distances

Other than the distance measures we obtained using the mixture model, some other metrics are selected for comparison, including two standard beta diversity metrics for any ecological distance-based measures, Manhattan and Euclidean distances, and three distance measures specific in microbiome analysis—Bray-Curtis measure, weighted, and generalized UniFrac distances. An unweighted UniFrac distance is not considered in this study since it does not contain taxa abundance information.

Let $x_{ij}$ and $x_{ik}$, for i=1,...,n, be the observed counts of OTU *i* in samples *j* and *k*, respectively. Let $b_{i}$ be the length of the branch *i* in a phylogenetic tree. The Euclidean distance is defined as

(6) $$D_{E}\left[j,k\right]=\sqrt{\sum_{i=1}^{n}{\left(x_{ij}-x_{ik}\right)}^{2}}.$$

The Manhattan distance is defined as

(7) $$D_{M}\left[j,k\right]=\sum_{i=1}^{n}\left|x_{ij}-x_{ik}\right|.$$

The Bray-Curtis distance measure is defined as

(8) $$D_{BC}\left[j,k\right]=\frac{\sum_{i=1}^{n}\left|x_{ij}-x_{ik}\right|}{\sum_{i=1}^{n}\left(x_{ij}+x_{ik}\right)}.$$

The weighted UniFrac distance is defined as

(9) $$D_{w}\left[j,k\right]=\frac{\sum_{i=1}^{n}b_{i}\left|x_{ij}-x_{ik}\right|}{\sum_{i=1}^{n}b_{i}\left(x_{ij}+x_{ik}\right)}.$$

And the generalized UniFrac distance is defined as

(10) $$D_{g(0.5)}\left[j,k\right]=\frac{\sum_{i=1}^{n}b_{i}\sqrt{x_{ij}+x_{ik}}\left|\frac{x_{ij}-x_{ik}}{x_{ij}+x_{ik}}\right|}{\sum_{i=1}^{n}b_{i}\sqrt{x_{ij}+x_{ik}}}.$$

Note that due to possible zeros in the denominator in Equations (8)–(10), we add a sufficiently small number ($1\times 10^{-8}$) in addition to the sum of observed counts. Sensitivity analysis was done and proved that adding a sufficiently small number in the denominator to avoid zeros does not affect the accuracy results.

### 2.3. Clustering Validation Indices

The clustering assessment utilizes the partition of data by quantifying the results of a clustering algorithm. The indices measure how well the clustering performed regarding both within and between clusters separability. Validation indices can be divided into internal indices and external assessments. When there are no standard labels of the data to evaluate the partition result, internal indices are considered as an assessment of the clustered data itself. Many internal validation indices have been proposed to choose the optimal number of clusters. The number of clusters is data-driven and is usually required to specify in advance by clustering algorithms. Approaches to select the optimal number for partition consider all possible choices that fit the algorithms and then find the best fit of the data after comparing indices. On the other hand, external assessment scores are calculated by directly comparing the partition results with the prior labels, given that the labels are not used in the model-building stage.

#### 2.3.1. Internal Validation Indices

Among the internal indices, similarities are observed in different indices measures. The Dunn index (DI) [41] is a metric for evaluating the separability of within clusters and between clusters. It is the quotient of the minimal distance between points of different clusters and the most substantial within-cluster distance. Let $C_{k}$ be a cluster of vectors. The diameter of the cluster, which is the largest distance separating two points in cluster $C_{k}$ is calculated by $\Delta_{k}=max_{i,j\in R_{k},i\neq j}\left|\right|S_{i}^{k}-S_{j}^{k}\left|\right|$. Consider $C_{k^{\prime }}$ as another cluster other than $C_{k}$. Let $\delta (C_{k},C_{k^{\prime }})$ be the inter-cluster distance metric for clusters $C_{k}$ and $C_{k^{\prime }}$. It is measured by the distance between their closest points:

(11) $$\delta \left(C_{k},C_{k^{\prime }}\right)=min_{i\in R_{k},j\in R_{k^{\prime }}}∥S_{i}^{k}-S_{j}^{k^{\prime }}∥,$$

where $S_{i}^{k}$ in cluster $C_{k}$ and $S_{j}^{k^{\prime }}$ in $C_{k^{\prime }}$. The Dunn index is defined as

(12) $$DI_{k}=\frac{min_{k\neq k^{\prime }}\delta \left(C_{k},C_{k^{\prime }}\right)}{max_{1\leq k\leq K}\Delta_{k}}.$$

#### 2.3.2. External Validation Assessment Measures

The results from clustering can be quantified by two measures: accuracy and Jaccard index. Both only consider the results obtained by the optimal number of clusters. Accuracy is how close the clustering results compared to the true cluster index. It is defined as the proportion of correctly clustered subjects. Note that when the clustered number of group *c* is less than the true number of clusters *k*, the accuracy of *c* groups are considered. When *c* is greater than *k*, a combination of *k* out of *c* groups with the highest accuracy is adopted. Jaccard index measures similarity between the clustered results and the original cluster labels which are defined as the ratio of the number of correctly classified subjects (intersection of predicted and real sets) to the number of the total sample size of the two groups (union of two sets):

$$J\left(C,K\right)=\frac{\left|C\cap K\right|}{\left|C\cup K\right|}.$$

### 2.4. Partitioning Algorithms for Clustering

Partitioning Around Medoids (PAM) algorithm introduced by Kaufman [20], as produced clustering results of this paper, is an adaption of K-means clustering, yet more computational efficient [42] and more robust to the random noises in the data [43]. The aim of clustering for pre-specified *K* groups is approached in the partitioning algorithm by incorporating two phases: initialization of *K* medoids and refinement of the initial medoids within clusters. The algorithm takes a greedy search technique in the first step to locate the *K* medoids in the data with the least computation. Then it uses a swap operation within the neighborhood in the second phase to minimize the objective function

$$\sum_{j\in C_{m}}d\left(m,j\right),$$

where $C_{m}$ is the cluster containing object *m* and d(m,j) is the sum of the distances from object *j* to the closest medoid *m*.

PAM follows steepest-ascent hill climber algorithm, which can be summarized as follows:

1. Initialize: randomly choose *K* of the *n* points in the dataset to be the initial cluster medoids.
2. Assign each data point to the closest medoid based on distance.
3. Refine: for each medoid *m* and non-medoid data point *j*, swap *j* and *m* and compute the total cost by the new medoid *j*. Select the best medoid in terms of minimum cost.
4. Repeat steps 2 and 3 until all the medoids are fixed.

**Figure 1:**
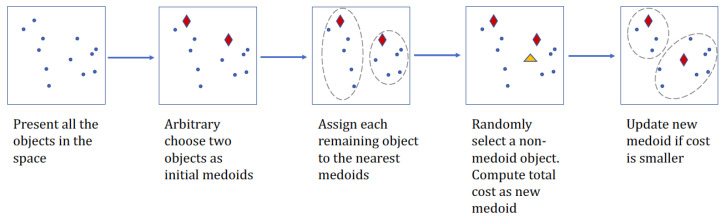
A flowchart to illustrate PAM algorithm for two clusters.

A flowchart can be found in Figure 1. PAM selects the points from the original data as medoids, which reduces the difficulty in explaining the cluster. Computation time can be saved by pre-calculating a distance matrix for all the data points. Then in the swap procedure for every iteration, scanning the matrix could be quickly done. In addition, as the refinement phase in step 3 is slow, re-calculation of the distance from only the points that have been moved between clusters to the new medoids in each iteration helps boost efficiency. For all the remaining points, distances can be re-used in objective function calculation.

The Algorithm 1 shows the detailed steps of the proposed clustering procedure using the mixture distribution.
**Algorithm 1:** for clustering based on joint mixture distribution:Specify L models for mixture models with components $G_{1},\ldots ,G_{M}$Bootstrap B datasets. For each bootstrap dataset, estimate and select the optimal model based on the distance between observed and expected aggregated countsCalculate weights for the selected model in each replicate and combine with weights for each component to obtain the final weights for the joint mixture modelEstimate the subject-specific mixture distribution and calculate the probability of a subject from each component in the mixture modelCalculate the distances using $L^{2}$ PDF and CDF normsCluster based on the distances using PAMCompare the scores from internal indices and select the optimal number of clusters as in the final algorithm

### 2.5. Simulation Studies

We conduct extensive simulation studies to evaluate the performance of the proposed algorithm with selected internal validation indices to finalize the optimal number of clusters for the clustering algorithm. To test how well the method performs in clustering, we derive the accuracy and the Jaccard index.

We simulate the data to mimic the OTU counts and their complex structure with class labels. We consider two scenarios with two sub-classes and three sub-classes, and each sub-class contains 200 subjects for total sample sizes of 400 and 600, respectively. All the results are replicated 100 times.

The simulation procedure for each OTU is as follows:

1. Determine the number of rates $r_{i}$ in each sub-class using $Beta(\alpha_{c},\beta_{c}).$
2. Generate the rate $r_{i}$ distribution in each sub-class from a mixture distribution with *M* components including a zero point mass and M−1 Gamma distributions. The number *M* is randomly chosen between 5 and 15.
3. Sample the number of rates from $M_{rate}∼Multinomial\left(P\left(M1\right),P\left(M2\right),...,P\left(M5\right),N_{c}\right)$ where $N_{c}$ is the sample size for sub-class *c*.
4. Sample $t_{i}$ for each subject from a Uniform(2/3,4/3).
5. Generate the observed count $n_{i}∼Poisson\left(r_{i}t_{i}\right)$.

For each scenario, 25 OTUs are simulated. The simulated count data contains three sets of zero proportions (ZP), first set with 13–27% zeros (low ZP) in each sub-class, second set with 39–61% zeros (medium ZP), and third set with 84–93% zeros (high ZP), to examine clustering performance under different ZP scenarios. ZP in every dataset is controlled by varying $\alpha_{c}$ in Beta distribution from Step 1. The details of the mixture distribution estimation can be found in Appendix B.

## 3. Results

### Simulation Results

**Figure 2:**
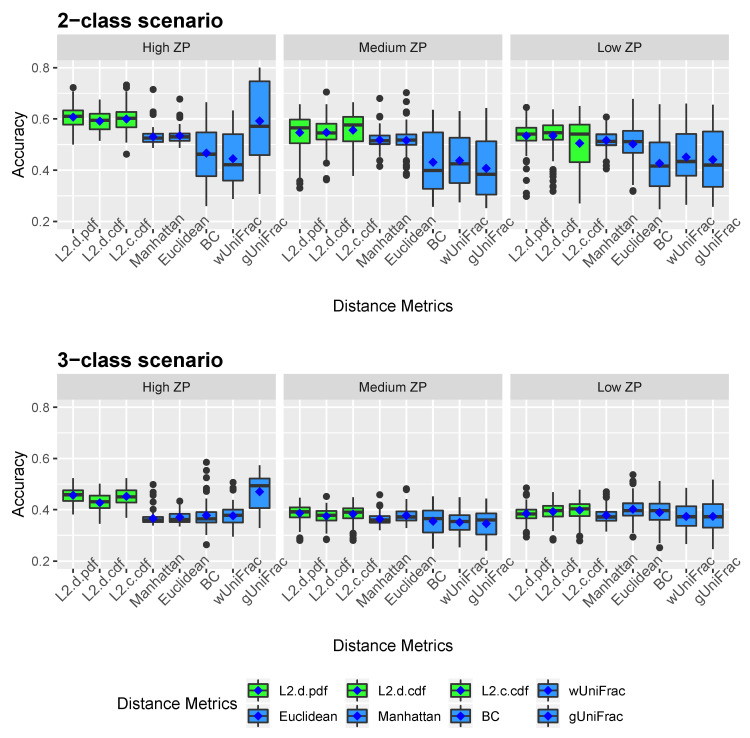
Accuracy boxplots for simulated data. Two-subclass and three-subclass scenarios are considered. Three different cases of proportion of zeros (ZP) are evaluated - high ZP, medium ZP, and low ZP, are presented in left, middle, and right, respectively. For each box of the boxplots, the center line represents the median, the two vertical lines represent the 25th percentiles to the 75th percentiles. The whiskers of the boxplots show 1.5 interquartile range (IQR) below the 25th percentiles and 1.5 IQR above the 75th percentiles. The mean are shown in blue diamond dots.

**Figure 3:**
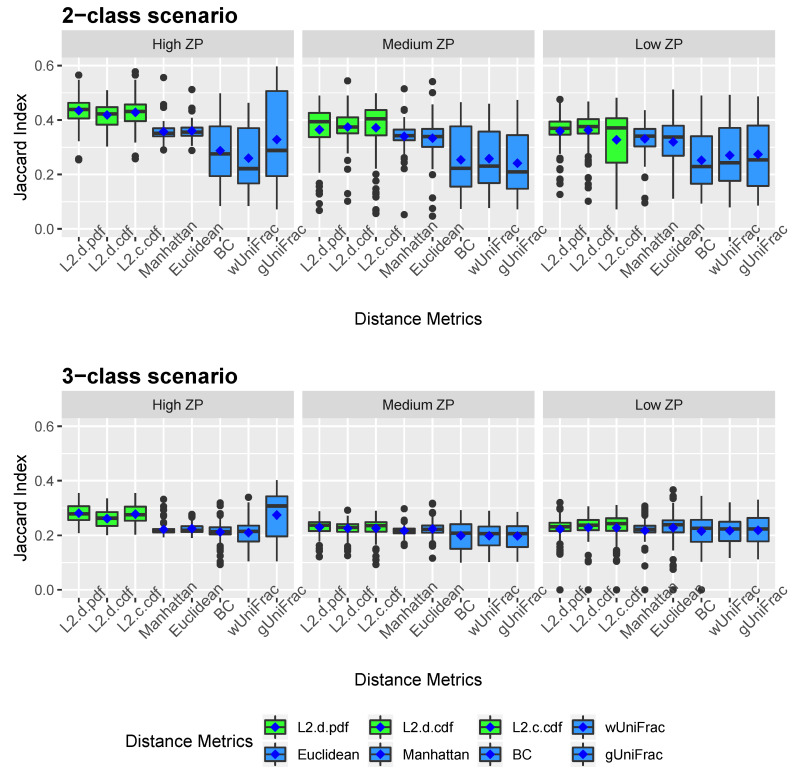
Jaccard index boxplots for simulated data. Two-subclass and three-subclass scenarios are considered. Three different cases of proportion of zeros (ZP) are evaluated - high ZP, medium ZP, and low ZP, are presented in left, middle, and right, respectively. For each box of the boxplots, the center line represents the median, the two vertical lines represent the 25th percentiles to the 75th percentiles. The whiskers of the boxplots show 1.5 interquartile range (IQR) below the 25th percentiles and 1.5 IQR above the 75th percentiles. The mean are shown in blue diamond dots.

Figure 2 and Figure 3 show the clustering results of simulated datasets under different scenarios with varying ZPs and number of sub-classes. The distance-based algorithm performance is evaluated through the accuracy and the Jaccard index and presented in boxplots. Specifically, *L2.d.pdf, L2.d.cdf, L2.c.cdf, Manhattan, Euclidean, BC, wUniFrac*, and *gUniFrac* represent the clustering results from a distance calculated by the mixture model using $L_{2}$ norms with discrete variable’s PDF, discrete variable’s CDF, continuous variable’s CDF, Manhattan distance, Euclidean distance, Bray-Curtis distance, weighted UniFrac distance, and generalized UniFrac distance, respectively. All the distances were calculated based on the relative abundance data. We conducted additional simulations to calculate the Manhattan, Euclidean, and Bray-Curtis distances after log-transformation. As we explained in methodology, since unweighted UniFrac distances neglect the abundance information and only consider presence/absence of species of branches in a phylogenetic tree, it is not included in the simulation studies. The top three boxplots in Figure 2 illustrate the accuracy of eight comparative distance metrics for high, medium, and low ZP scenarios when the simulated dataset contains two sub-classes. The bottom three boxplots are the accuracy of the 3-subclass simulation scenario. Our proposed distance measures are marked in green as opposed to the other distance metrics in blue. Jaccard index boxplots (Figure 3) are constructed in the same way as in Figure 2. Mean accuracy (MA) and mean Jaccard index (MJI) are shown in Table 1, calculated by averaging the 100 replicates results in each scenario.

We observed that by implementing the proposed distance measures in the clustering algorithm, both accuracy and Jaccard index outperform the results by other distance metrics, especially when the datasets contain a substantial amount of zeros. Clustering using the three proposed $L_{2}$ norms in both 2-subclass and 3-subclass scenarios has considerable improvements with the increase of zero proportions in the datasets. The mean accuracy calculated based on 100 replicates achieves around 0.6 for the proposed $L_{2}$ norms under high ZP design in 2-subclass scenarios and 0.45 in 3-subclass scenarios. For scenarios with fewer zeros, the $L_{2}$-norm distance measures have competitive clustering performance as the competing distance metrics. Among the three $L_{2}$ norms, the $L_{2}$ discrete PDF distance has better clustering performance across ZP settings. Out of six settings that we investigated in the 3-subclass scenario, the generalized UniFrac distance and the Manhattan distance with log-transformation provide the best partition with a high and low proportion of zeros, respectively. In contract, the $L_{2}$ norms show advantages in terms of MA and MJI in the rest of scenarios. Noticeably, the generalized UniFrac distance shows a large variability in the estimation of accuracy. Overall, Manhattan, Euclidean, Bray-Curtis, and weighted UniFrac distance metrics do not distinguish the proportion of zeros in 2-subclass datasets and provide close to the random guess accuracy of 0.5 in 2-subclass scenarios. Jaccard index reveals a similar pattern as accuracy.

The average number of clusters over all the iterations are presented in Table 2. The $L_{2}$-D CDF norm predicts the number of clusters the most accurate in 2-subclass scenarios. On the other hand, generalized UniFrac distance and Manhattan distance with log transformation have closer prediction to the actual cluster numbers in the 3-subclass scenarios. However, Bray-Curtis, weighted UniFrac, and generalized UniFrac distances overestimate number of clusters dramatically in the two-subclass situations. Thus, the results in three-subclass scenarios are doubtful to some extent.

## 4. Real Data Implementation

To demonstrate how well our proposed method works, we analyze the data from Hill-Burns et al. [1] that relates the gut microbiome to Parkinson’s disease (PD). The dataset contains stool samples of 197 PD cases and 130 controls. 16S rRNA amplicon sequencing of DNA was extracted for microbial composition performed by Illumina MiSeq. OTUs were picked using a reference of Greengenes 16S rRNA gene sequence database [44] at 97% similarity released in August 2013. The study has shown the association between the dysbiosis of gut microbiome and PD. Besides, the case-only analysis identifies a significant interaction effect between the microbiome and PD medications, including catechol-O-methyl transferase (COMT) inhibitors, anticholinergics, and carbidopa/levodopa.

We apply our algorithm to the PD cases to explore the sub-populations of the PD using gut mirobiome data. The pre-processing step is done by including OTUs on the genus level and excluding ones with the probability of zero relative abundances higher than 80%, resulting in a total of 280 OTUs used for all the samples in our analysis. We compare the proposed $L_{2}$ norms with the other three distance metrics with and without log-transformation on the relative abundance data. Various internal indices are applied to distance measures for sensitivity analysis. Selection results of the number of clusters are illustrated in Table 3. The maximum number of clusters is set to ten, meaning that the optimal number is between 2 and 10. Different combinations of distances and internal indices provide moderate variations. For Dunn and Xie-Beni [45] indices, $L_{2}$ norms tend to cluster the data into two or three subgroups while in both with and without log-transformation situations, Manhattan, Euclidean, and Bray-Curtis metrics prefer more clusters except for non-transformed Euclidean distance. Wemmert-Gancarski index provides fewer subgroups than the others across the distance measures. No profound trend is found for the Silhouette index.

To illustrate the clustering algorithm, the $L_{2}$-D PDF norm is selected as an example for further analysis. We explore OTUs between two clusters for the dataset, and the top 5 significantly different OTUs between clusters are *Akkermansia*, *Anaerotruncus*, *Bacteroides*, *Anaerococcus*, and *Akkermansia*. Among these OTUs, *Akkermansia* [1,46] has previous reported associations. The characteristics of the five OTUs are summarized in Table 4.

## 5. Discussion

We simulate six different scenarios to evaluate the performance of the proposed method thoroughly, using the accuracy and the Jaccard index to reflect clustering results, considering different zero proportions under 2-subclass and 3-subclass settings. Both the accuracy and the Jaccard index are improved or competitive compared to other distance metrics, suggesting better separation among subgroups. Our method performs the best in high and medium zero proportion scenarios, therefore, it is recommended to use our clustering algorithm when a large number of zeros presenting in the data. Under the PAM framework, all distance matrices (Manhattan, Euclidean, Bray-Curtis, and UniFrac) can be used as inputs for clustering. However, as shown in our simulation studies, the pairwise distances calculated by the mixture model perform better than the other distance matrices under a variety of scenarios.

The clustering algorithm involves multiple options, such as the choice of distance measures, the internal indices to specify the number of clusters, and the approach to clustering. Due to a lack of widely accepted standardization, making different choices at each step may lead to various outcomes. Many choices are available regarding the selection of the number of clusters. The decision to make about the optimal number of clusters each time highly relies on data structure, thus case-specific. As we choose to use the Dunn index as internal validation indices for simulation studies, sensitivity analysis is performed using other internal indices. All the considered indices are also compared in real data. Our algorithm classifies subgroups among the PD cases and presented the ability to identify statistically significant distinct OTUs which have association with PD.

The proposed method focuses on distance-based clustering. The next step is to perform partition based on models such as Dirichlet-multinomial and compare with our method. We will also explore the possibility of extending the proposed method to adopt longitudinal trajectories of subjects for deep insights into the dynamic biological mechanisms. The proposed method could be easily extended to high dimensional data with overdispersion. Besides that, we are working on the extension of this proposed method on other microbiome and disease correlation data.

As all clustering methods, one limitation of this algorithm is that suitable internal indices are hard to select for every new data. Thus an optimal and robust number of clusters is difficult to obtain. Besides, for the $L_{2}$-norm distances, variable selection is not possible to be developed in clustering. Nevertheless, the proposed algorithm incorporates ad-hoc distance for microbial sequencing data, which provides effective clustering and broader vision to investigate the connection between the microbiome and human health. The introduced clustering algorithm can be seen as a good additional tool for the analysis of microbial data besides the currently used methods.

## 6. Conclusions

In this article, we propose a distance-based unsupervised machine learning method to cluster subjects based on their microbial structure. We show that our method provide funtional partitions among subjects under various scenarios in simulation studies, and we apply it to a gut microbiome dataset for Parkinson’s disease. The distance measures we adopted in the clustering algorithm are capable of capturing the underlying rate distributions of microbial counts, through mixture distributions which take account to zero inflation and overdispersed values. $L_{2}$ norms are calculated based on the mixture distributions’ PDF and CDF, respectively, and further used in partition around mediod for clustering.

## Figures and Tables

**Table 1:** Mean accuracy (MA) and mean Jaccard index (MJI) estimations. A bold value represents the best cases under each scenario. Distances calculation was conducted on the simulated data inputs with and without log-transformation: Manhattan_log, Euclidean_log, Bray-Curtis_log were models using log-transformation, while $L_{2}$-D PDF, $L_{2}$-D CDF, $L_{2}$-C CDF, Manhattan, Euclidean, Bray-Curtis, weighted UniFrac, and generalized UniFrac were without log-transformation.

**Two-Subclass Scenarios**						
	**High ZP**		**Medium ZP**		**Low ZP**	
**Distance**	**MA**	**MJI**	**MA**	**MJI**	**MA**	**MJI**
$L_{2}$-D PDF	**0.608**	**0.435**	0.547	0.365	**0.534**	0.359
$L_{2}$-D CDF	0.591	0.419	0.547	**0.374**	**0.534**	**0.363**
$L_{2}$-C CDF	0.600	0.428	**0.556**	0.371	0.505	0.327
Manhattan	0.530	0.357	0.518	0.340	0.516	0.331
Euclidean	0.530	0.357	0.518	0.341	0.516	0.331
Bray-Curtis	0.467	0.288	0.434	0.258	0.427	0.253
Weighted UniFrac	0.445	0.260	0.437	0.258	0.451	0.270
Generalized UniFrac	0.605	0.420	0.407	0.241	0.441	0.274
Manhattan_log	0.534	0.360	0.520	0.334	0.499	0.317
Euclidean_log	0.534	0.360	0.516	0.333	0.502	0.320
Bray-Curtis_log	0.467	0.287	0.431	0.254	0.427	0.252
**Three-Subclass Scenarios**						
	**High ZP**		**Medium ZP**		**Low ZP**	
**Distance**	**MA**	**MJI**	**MA**	**MJI**	**MA**	**MJI**
$L_{2}$-D PDF	0.456	**0.281**	**0.386**	**0.230**	0.373	0.223
$L_{2}$-D CDF	0.427	0.261	0.375	0.226	0.381	0.228
$L_{2}$-C CDF	0.452	0.277	0.383	0.226	0.386	0.228
Manhattan	0.366	0.222	0.364	0.217	0.367	0.217
Euclidean	0.375	0.229	0.364	0.220	0.384	0.234
Bray-Curtis	0.379	0.211	0.348	0.193	0.369	0.207
Weighted UniFrac	0.376	0.210	0.351	0.198	0.374	0.217
Generalized UniFrac	**0.470**	0.274	0.346	0.197	0.374	0.219
Manhattan_log	0.383	0.234	0.379	0.227	**0.404**	**0.243**
Euclidean_log	0.371	0.225	0.377	0.224	0.390	0.228
Bray-Curtis_log	0.378	0.212	0.355	0.199	0.378	0.215

**Table 2:** Average number of clusters for all simulation scenarios. Optimal number of clusters in each replicate is calculated by Dunn internal indices. A bold value represents the closest estimation of number of clusters to the ground truth.

	Two-Subclass Scenarios			Three-Subclass Scenarios		
Distance	High ZP	Medium ZP	Low ZP	High ZP	Medium ZP	Low ZP
$L_{2}$-D PDF	2.47	2.73	2.30	2.48	2.74	2.35
$L_{2}$-D CDF	**2.26**	**2.28**	**2.28**	2.16	2.43	2.30
$L_{2}$-C CDF	2.40	2.46	2.65	2.26	2.88	2.63
Manhattan	2.87	2.73	2.84	2.48	2.68	2.63
Euclidean	2.86	2.72	2.85	2.93	2.85	2.91
Bray-Curtis	3.08	3.47	3.44	3.31	3.57	3.44
Weighted UniFrac	3.38	3.39	3.23	3.35	3.36	3.29
Generalized UniFrac	2.88	3.39	3.16	**3.01**	3.34	3.20
Manhattan_log	2.72	2.95	3.11	2.82	**2.91**	**2.98**
Euclidean_log	2.76	2.94	3.05	2.46	2.80	2.78
Bray-Curtis_log	3.08	3.50	3.44	2.80	3.48	3.17

**Table 3:** Mean with standard deviation and median for OTUs that are significantly different (p< 0.001) between two clusters using Dunn internal indices and $L_{2}$-D PDF distance.

Distance	Dunn	Silhouette Index	Wemmert-Gancarski	Xie-Beni
$L_{2}$ discrete PDF	2	7	2	2
$L_{2}$ discrete CDF	3	2	2	3
$L_{2}$ continuous CDF	2	5	3	3
Manhattan	10	4	4	10
Euclidean	3	3	3	3
Bray-Curtis	7	10	2	10
Manhattan_log	9	2	10	10
Euclidean_log	5	2	5	5
Bray-Curtis_log	9	9	2	10

**Table 4:** Optimal number of clusters for distance metrics by various internal indices.

OTU	Full Sample (n = 197)	Cluster 1 (n = 166)	Cluster 2 (n = 31)
**g_Akkermansia**			
Mean (sd)	404.3 (956.8)	479.7 (1025.2)	0.9 (1.4)
Median (Min,Max)	1 (0,5284)	3.5 (0,5284)	1 (0,7)
**g_Anaerotruncus**			
Mean (sd)	4.5 (11.8)	4.2 (12.5)	6.2 (7.2)
Median (Min,Max)	0 (0,120)	0 (0,120)	3 (0,27)
**g_Bacteroides**			
Mean (sd)	0.6 (1.5)	0.5 (1.4)	1.3 (1.8)
Median (Min,Max)	0 (0,9)	0 (0,9)	0 (0,5)
**g_Anaerococcus**			
Mean (sd)	8.8 (40.5)	8.8 (43.6)	8.8 (16.3)
Median (Min,Max)	0 (0,352)	0 (0,352)	2 (0,79)
**g_Akkermansia_**			
Mean (sd)	198.8 (811.5)	0.7 (1.9)	1259.8 (1709.5)
Median (Min,Max)	0 (0,6278)	0 (0,17)	378 (53,6278)

## Abbreviations

The following abbreviations are used in this manuscript:

OTU	operational taxonomic unit
BC	Bray-Curtis distance
JS	Jenson-Shannon distance
PAM	partition around medoids
DI	Dunn index
MA	mean accuracy
MJI	mean Jaccard index
PD	Parkinson’s disease

## Appendix A. Mixture Model

### Appendix A.1. Individual Mixture Distribution Estimation

A non-parametric bootstrap is adopted to estimate a mixture model with a practical consideration to avoid overspecified and overfitted bootstrap data. The selection of the best model among all bootstrap datasets is unnecessary since the final model will be a set of weighted optimal models from all the datasets so that every component can be incorporated in the estimation. Define a set of models $\Psi_{l},l=1,\ldots ,L$ with different mixture components combination. Sample *B* subsets from the original data and estimate weights ${\vec{w}}_{bl}$ for each model *l* and subset *b*. In each bootstrap dataset, the estimated aggregated counts ${\vec{\hat{y}}}_{l}=\sum_{k}{\hat{y}}_{kl},k=0,\ldots ,C,C+$ for model *l* are compared with the observed aggregated counts $\vec{y}$ through the distance $D(\vec{y},{\vec{\hat{y}}}_{l})$ for l=1,…L and the best model $l_{b}^{*}$ with minimum distance is chosen in subset *b*. The selected optimal model has weight $v\left(l\right)=\sum_{b}I\left(l_{b}^{*}=l\right)/B$, and the weight for each component in the mixture distribution is $w_{m}=\sum_{l}v\left(l\right)w_{ml}$.

Once we have an estimate of the mixture component weights $\vec{w}$ for the final set of mixture components $G=(G_{z},G_{1},G_{2},\ldots ,G_{M},G_{h})$, the individual-specific mixture probabilities condition on $\vec{w}$ can be obtained for each component. Specifically, the probability of observing counts *k* from subject *i* being from each mixture component $G_{q}$ can be written as

(A1) $$P\left(i\in G_{q}\right)=\left\{\begin{array}{cc} I(k=0), & \mathrm{if}\;q=z\\ w_{q}\frac{\Gamma (k+\alpha_{q})}{\Gamma \left(k+1\right)\Gamma \left(\alpha_{q}\right)}{\left(\frac{\beta_{q}}{t_{i}+\beta_{q}}\right)}^{\alpha_{q}}{\left(1-\frac{\beta_{q}}{t_{i}+\beta_{q}}\right)}^{k}, & \mathrm{if}\;q=1,...,M\\ I(k>C), & \mathrm{if}\;q=h \end{array}\right.$$

Denote the individual-specific mixture weights as $\vec{w_{i}}=\left(w_{iz},w_{i1},\ldots ,w_{iM},w_{ih}\right)$, and weights are calculated as $w_{iq}=P\left(i\in G_{q}\right)/\sum_{q}P\left(i\in G_{q}\right)$. Since the model components G remains the same for all the samples, the individual-specific mixture weights become the only variation to compute pairwise distances among samples. Hence distances are calculated based on two parts, individual weights and the probability of an observed count through Poisson-Gamma mixture probabilities $NB(\alpha_{q},\frac{\beta_{q}}{1+\beta_{q}})$ from mixture component $G_{q}$. The probability of observing count *k* from mixture component $G_{q}$, q=z,1,...,M,h is

(A2) $$P_{G_{q}}\left(k\right)=\left\{\begin{array}{cc} I(K=0), & \mathrm{if}\;q=z\\ P_{NB}\left(K=k|\alpha_{q},\beta_{q}\right), & \mathrm{if}\;q=1,...M\\ I(K>C), & \mathrm{if}\;q=h \end{array}\right.$$

Note that the distribution can estimate structural and non-structural zeros separately, where $P_{G_{z}}\left(0\right)=w_{z}$ is for structure zeros and $P\left(k=0\right)=\sum_{q}P_{G_{q}}\left(0\right)-w_{z}$ is for non-structure zeros.

## Appendix B. Simulation

### Appendix B.1. Mixture Distribution Estimation

The underlying mixture distributionis modeled with pre-set four parts to estimate the simulated data.

- Low rate part: set five Gamma distribution Γ(1,2),Γ(1,1),Γ(2,1),Γ(3,1),Γ(4,1), and five models, with the first model including all the distributions, the second model including the last 4 distributions, until the fifth model including only the last Gamma distribution;
- Medium rate part: one model with four Gamma distribution Γ(5,1),Γ(6,1),Γ(7,1),Γ(8,1);
- Higher rate part: one model with three Gamma distribution Γ(11,1),Γ(18,1),Γ(19,1); each α value in Gamma distribution is chosen by uniformly binned the OTUs on a log-transformed scale from 8% to the 85% quantile;
- High count part: a point mass which combines all the counts greater than the 85% quantile.

The estimation of the joint model is replicated through 300 bootstraps. The estimates of weights in the model are obtained by minimizing the least squares objective function using the Broyden- Fletcher-Goldfarb-Shanno algorithm [47]. We used R packages *NLoptr* [48] for the model estimation, and *cluster* [49] and *clustCrit* [50] for comparison of clustering performance between distance measures.

The appendix is an optional section that can contain details and data supplemental to the main text. For example, explanations of experimental details that would disrupt the flow of the main text, but nonetheless remain crucial to understanding and reproducing the research shown; figures of replicates for experiments of which representative data is shown in the main text can be added here if brief, or as Supplementary data. Mathematical proofs of results not central to the paper can be added as an appendix.
